# Progress in Medicine

**Published:** 1896-07-25

**Authors:** 


### Progress in Medicine.

### DISEASES OF THE CIRCULATORY SYSTEM.

(*Continued from page* 263.)

**Arterio-Sclerosis.**—Some important facts are brought before our notice by Adami,[5] in his Middleton lectures at M'Gill University. Just as the heart muscle and pericardium are nourished by the coronary vessels, so the media and adventitia of the arteries are nourished by the vasa-vasorum; whereas in health the intima appears to be non-vascular, and is probably nourished directly by the blood circulating in the vessels. The diseases to which the arterial walls are subject are closely comparable with those of the heart. Inflammatory affections may possibly arise, but, at any rate, obviously inflammatory conditions are relatively rare. The most common form of arterial disease is arterio-sclerosis or atheroma. Accepting Councilman's classification of atheroma under three heads—(*a*) a nodular arterio-sclerosis, (*b*) senile arterio-sclerosis, and (*c*) diffuse arterio-sclerosis—he also acknowledges that these three merge into one another; but he considers in his lecture the nodular form in particular, the true endarteritis nodosa of Virchow. The process begins by the development of semi-transparent, almost gelatinous, plaques here and there upon the walls of the aorta and larger vessels; later, the plaques are firm, dense, or may even be calcareous. We know that (1) the endothelium over the plaque is continuous and apparently unaffected; (2) the new tissue is laid down regularly in layers parallel to the endothelium; (3) the connective tissue of the intima in the immediate neighbourhood of the plaque passes imperceptibly into the hypertrophied connective tissue forming the plaque; (4) the oldest layers of the plaque are evidently those nearest to the elastic lamina, the most recent are those beneath the endothelium and farthest away from the vasa-vasorum of the media; and (5) the plaque is devoid of vessels save and except in those cases in which there is an evident attempt at the removal of necrosed atheromatous material, and vessels penetrate into the atheromatous mass from the media in a manner comparable with their passage into a thrombus. Such passage, it will be recognised, is of a secondary nature. The picture presented is, then, not one usually associated with inflammation, but rather what one would expect to find in an ordinary connective tissue hypertrophy, while the degeneration appearing in the deeper layers is what might be expected to occur in a non-vascular area in which layer after layer of connective tissue cut off these deeper parts from their ordinary source of nutrition. This, however, is not the whole picture. Constantly accompanying, and indeed preceding, the changes in the intima, there is to be recognised an injury or degeneration of the outer coats. The muscle fibres of the media frequently exhibit hyaline, and other degenerative changes; and there may be some replacement fibrosis, or again evidences of more extensive failure of nutrition and of necrobiosis in the shape of small areas of calcareous deposit. That is to say, the media is very definitely affected, and, as Thoma's experiments have fully proved, the plaque of overgrowth of the intima corresponds to a localised giving way of the arterial wall, to a localised slight bulging of the same; for, by injecting affected arteries with paraffin under a pressure of 160 millimetres of mercury, Thoma obtained a smooth cylindrical mould, showing no signs of depressions corresponding to any projecting plaques, thus the hypertrophied intima fills and obliterates the slight bulgings of the outer coats. However produced, there can be no question that we have here to deal with a compensatory hypertrophy of the intima. In the diffuse form, also, Thoma has demonstrated that the growth of connective tissue in the intima has a similar compensatory nature. Is this process to be denominated an inflammation? Were the main nutrition from the vasa-vasorum of the media, we should find following disturbances in these vessels that wedges of degeneration extending towards the lumen occurred, instead of the broad plaques that actually arise. Alterations in the vasa-vasorum failing to explain the new growth, Adami falls back upon altered tension, as a factor to be adduced in partial explanation of these cases of increased growth. With Thoma, we may possibly also call in the agency of the sensory and trophic nerves as governing the growth; but here we enter further upon speculative ground. They may play, they probably play (says Adami) an active part, but we have no direct evidence that they do. Adami regards the overgrowth as functional, and that it is brought about by the conditions of the circulation, not of the circulating fluid.

**Affections of the Myocardium.**—Idiopathic hypertrophy is classified by Whittaker[6] according to its etiology as follows: (1) Hypertrophies, due to obstacles in the heart itself, *e.g.*, valvular disease; (2) hypertrophies, due to increased vascular resistance; (3) hypertrophies, due to disease of the heart's muscle itself; (4) hypertrophies, due to affections of the nervous system. During pregnancy the heart increases in size and weight, due to the increased work thrown on the heart. Moreover, there is an actual increase in the amount of blood during pregnancy. Anything which favours the development of arterio-sclerosis also precipitates the changes of age, *e.g.*, alcoholism, syphilis, Bright's disease, diabetes, gout, saturnism, and psychical influences. Hypertrophy from irritation of the vagus, or that occurring in Graves' disease, are examples of that due to nervous influence.

**Acute Endocarditis.**—A very interesting observation has been made by Dauber and Borsh[7] in a case of malignant endocarditis following gonorrhœa. Ten days after the urethral discharge was first noticed symptoms of general infection appeared, viz., remitting fever with pains in joints, muscles, &c., and later valvular carditis. Post-mortem the aortic valves were found extensively ulcerated. Now the important point lies in the fact that the aortic vegetations consisted of cocci, cells, and fibrin, and the cocci, which when isolated appeared as diplococci, and in size, appearance, and staining reactions were indistinguishable from gonococci, yet on culture proved not to be gonococci. Aware of Lustgarten and Mannaberg's discovery that there occurred in the normal urethra diplococci that could only be differentiated by culture colonies from gonococci, Wilmi inferred that the endocarditis following gonorrhœa depended not on gonococci but on other diplococci, which the action of the gonococci in the urethra enabled to enter the blood, and he demonstrated the presence of the diplococci in the urethra but not on the cardiac vegetations. Dauber and Borsh have proved that in their case the endocarditis were not caused, but they guard us from inferring that in every case of endocarditis associated with gonorrhœa the cause is a coccus other than the gonococcus. In Leyden's and in Councilman's case the observers considered that they had found gonococci in the vegetations, but cultures were not made. It is noteworthy that salicylic acid and phenacetin did not prevent the joint pains and general symptoms in Dauber and Borsh's cases.

Kelynack[8] has collected and analysed twenty-five post-mortem cases of malignant endocarditis occurring at Manchester Royal Infirmary during the last four years. The twenty-five cases gave a percentage of 3 compared with all other medical cases examined during the same period, 68 per cent. being males and 32 per cent. females. The average age of males, 32; of females, 26. A distinct history of rheumatism was obtained in nine of the cases, but in several others there were strong grounds for believing patients to have been the subjects of rheumatic processes, and in 20 there was distinct evinence of old cardiac disease, and in four only were there adequate grounds for considering the malignant endocarditis a primary affection. In only one case was the malignant endocarditis limited to the right side of the heart, in three both right and left were involved, the tricuspid and mitral in one, and the tricuspid, mitral, and aortic in two. In six the mitral was the only valve affected. In three the aortic valve was alone affected, but in two others the left ventricle was also involved as well as the aortic. In six both mitral and aortic valves were the seat of malignant endocarditis, and in another case vegetations were found in the aorta itself, as well as in mitral and aortic valves. In only one case were the pulmonary valves involved, and then vegetations were also found in the pulmonary artery and right ventricle. Among the secondary and associated lesions Kelynack mentions that pneumonia was present in ten instances, being in the majority of cases secondary. Acute pleurisy was noted in four, and effusion in three cases; acute pericarditis in two, effusion in three, and adhesion in three. Splenic enlargement was a special feature in nineteen cases. Nephritis, chiefly acute parenchymatous, was present in four.

**Tuberculous Endocarditis.**—Leyden[9] found tubercle bacilli in four cases of recent warty endocarditis. In one case the bacilli were not only present in the vegetations, but also in ante-mortem thrombi. The bacilli were largely in the cells, and Leyden thinks that the bacilli do not enter the cells while in the vegetations, but are transported by the cells, so that the latter, so far from protecting the body against infection, favour the spread of the bacilli.

**Infective Endocarditis in Tonsilitis** seems to have been due to the staphylococcus aureus in a case recorded by Charrin.[10] A male aged eighteen, with temperature 104 deg. Fahr., had dyspnœa, with broncho-pneumonic patches in the right lung, liver and spleen somewhat enlarged, slight albuminuria and muffled heart-sounds, and the right tonsil swollen. The diagnosis was infective broncho-pneumonia, possibly influenzal, following tonsilitis. Post-mortem, in the broncho-pneumonic patches and in the valvular vegatations the staphylococcus aureus was present, the same microbe having been found in swarms during life in the tonsil. These are the same micro-organisms that Bouchard isolated from the lesions of chronic and subacute rheumatism, so often preceded by tonsilitis.

**Crystallised Digitalin.**—Nativelle's crystallised digitalin has been employed by Edgren.[11] He found that (1) crystallised digitalin, in doses of a milligramme, exercises a tonic action on the heart, rendering it more regular, and giving diuretic effects superior to the action of the infusion of digitalis; (2) this action manifests itself with certainty in every case, but, it goes without saying, in variable degree; (3) it never produced symptoms of digitalis intoxication.

**Coronilla,** according to a recent writer,[12] acts like digitalin in causing death by arresting the heart in systole. If the dose is a little smaller it diminishes the arterial pressure, the pulse becomes irregular, and death supervenes. Toxic doses increase the blood pressure while producing diuresis, and slacken the cardiac beats. Coronilla increases the force of the heartbeats, diminishes œdema, increases diuresis, and ameliorates dyspnœa, but it has no influence at all when the muscular substance of the heart is degenerated. It is said not to cause vomiting or diarrhœa. It may be prescribed as follows: (1) The extract of coronilla, in doses of from 6 to 15 grains a day. (2) The alcoholic tincture, in potions containing from 45 to 60 grains. (3) Coronilla, in doses of from 3 to 4 grains a day. The preparations of coronilla should be given in small divided doses during the day.

**Theobromine** was isolated in 1882 by Woskessenski. Huchard[13] has used it for two years past in various affections of the heart and kidney, as a diuretic. Theobromine appears to act by a direct and elective action on the renal epithelium. It is important to determine whether in healthy individuals its more or less prolonged use does not determine prolonged stimulation of the kidney so as to cause albuminuria, and whether in albuminurics theobromine augments the albuminuria. Huchard has found repeatedly that while in cases where no albumen was present in the urine before the administration of theobromine none appeared during its exhibition, yet in chronic albuminuric cases the amount of albumen was increased.

Strophanthus is now a well-known cardiac tonic, but its very general use only makes the recent observations of George Balfour[14] the more important and acceptable. He is of opinion that the remarkable contrast between the action of strophanthus and digitalis have not been sufficiently emphasised. It is not enough that in a series of cases the employment of the drug was followed by the disappearance of dropsy, and that the pulse tracings showed an apparent improvement in the pulse-rate and in the blood-pressure, for this has happened without the use of either digitalis or strophanthus. The heart works so well within its power that even when there is some valvular incompetence there is not a falter in the action of the heart, nor any indication in the pulse-tracing of any interruption of the equability of the blood-pressure—at any rate, not for many a long day. But whenever, from overwork, privation, or debilitating illness, the general metabolism is interfered with, the heart suffers, its function is impaired. Like every weak muscle, the enfeebled myocardium reacts to stimuli irritably and irregularly, and does its peculiar work imperfectly. If we put such a patient to bed, we can readily understand that this rest is sufficient to give such a fillip to the general metabolism as enables the heart to recover its tone and to discharge its function more perfectly. Now, rest is of first importance, though recovery from failing heart may be aided by the judicious use of purgatives, of diuretics, or of such drugs as either diminish the work of the heart or restore the tone of the myocardium. It is evident that those drugs alone are of paramount value which increase the elasticity of the myocardium, and that this action can only be permanent when accompanied by a corresponding improvement in the general metabolism. The action of digitalis purpurea is in moderate doses to improve the elasticity of all muscular fibre, and as all the blood passes many times through the heart for once that it passes through any other muscle, the myocardium is powerfully affected, while the other muscles remain practically uninfluenced. In like manner the muscles of the arterioles are also early and powerfully affected, though not to the same extent as the heart, as only the blood going to the district supplied by these vessels passes through them. The increased elasticity imparted to the myocardium by digitalis enables it to expand and to contract more perfectly, and as, owing to the increased elasticity of the muscles of the arterioles, the arteries empty themselves more slowly the blood accumulates within them, the blood pressure gradually rises, and, in accordance with Marey's law, the heart's action is slowed. No doubt this is partly due to the action of digitalis on the vagus nerve, but the action on the muscles is sufficient to account for all the phenomena observed, and it is with it we are chiefly concerned. The result of this rise in the blood pressure is that the secretions are improved and all the tissues of the body, amongst them the myocardium, are fed with richer blood at a higher pressure, so that metabolism is more perfect and every function is more efficiently performed. As the blood accumulates within the arteries the veins are correspondingly emptied, the serous soakage of the tissues is reabsorbed, and the excess of water that thus gets into the blood is removed by the kidneys, so that for a time the urine is increased. The improved metabolism of the myocardium enables it to discharge its functions more perfectly and to resist successfully all those deteriorating influences to which a weak heart succumbs.

All the benefits we obtain from digitalis are inseparably connected with its tonic action, especially its action on the myocardium. Digitalis is no opium to the heart; it does not relieve by narcotising, but it soothes cardiac irritability by strengthening the cardiac muscle. These benefits are readily obtained by very moderate doses of the drug, though great benefit may at times be obtained more rapidly by the judicious administration of larger doses; yet the long continuance of even small doses is often followed by the very best results, while the abuse of the drug, so frequently accompanied by distressing, if not alarming, symptoms, proceed upon an entire misconception of the true action of digitalis. The action of strophanthus hispidus is so entirely different from that of digitalis that it is difficult to see how any comparison can be made between the two. Its action is twofold: in small doses it arrests the heart in diastole, and in large doses it arrests it in systole, the former action being that which alone is useful therapeutically; in it the diastole is prolonged, and the contractile energy of the heart is at the same time increased. The type is thus of bradycardia, a slow-beating heart sending forward a large blood-wave with each contraction—not a type of heart usually associated with much vigour of the circulation. Strophanthus acts three thousand times more powerfully on the heart than digitalis; so that if some still dread digitalis as a dangerous cardiac poison, what must their feelings be towards strophanthus? Indeed, whether we regard the uncertainty of its direction or its excessive energy as a poison, it seems difficult to formulate a more forcible indictment against any drug than that presented by a bare statement of the facts recorded against strophanthus by its most able investigator and its most ardent advocate. Further, while strophanthus acts 3,000 times more powerfully on the heart than digitalis, it acts 100 times less powerfully than digitalis upon the muscles of the arterioles. From the entire absence of any appreciable action by strophanthus on the arterioles, the blood flows freely from the arteries into the veins, and any little rise of blood-pressure there may be is entirely due to the systole of the heart. The typical (therapeutically induced) strophanthus heart has a prolonged diastole, so that the arteries have a longer time than usual to empty themselves, for the ventricle has been filling during this period, its systole sends an unusually large blood wave into unusually empty arteries—precisely what happens in a case of bradycardia. So evanescent a rise in blood-pressure can have no appreciable effect on either general metabolism or on that of the myocardium. Herein the action of strophanthus differs most essentially from that of digitalis, and from the absence of any improvement in the metabolism of the myocardium the stimulating action of strophanthus must tend still further to exhaust it instead of strengthening it.

The tendency to arrest in diastole is not due to any action on the cardio-inhibitory apparatus, because paralysis of the intracardiac terminations of the vagus by atropine does not prevent its occurrence. Balfour explains the diastolic type of action as due to exhaustion of the heart. When death occurs in fatal systole after the administration of strophanthus, the cardiac muscle is acid, it is fatally poisoned, it has been forced into fatal systole. The smaller doses resulting in death with diastole act by gradually over-stimulating and exhausting the heart muscle. It may be fatal without warning. The action of strophanthus is like that of all its congeners of the apocynaceæ, and is that of a cardiac poison, and not a cardiac tonic. While in large doses it forces the heart into a fatal systole, in small doses it stimulates the heart to increased action, and by calling on its reserve of energy without improving its metabolism, it causes death in diastole from exhaustion, and the more feeble the heart the greater the risk attending this peculiar action.

**The Anacrotic Pulse.**—The anacrotic pulse has been noticed by Huchard[15] on four occasions, and only in young subjects with very marked aortic valve stenosis. The characteristics of the anacrotic pulse are as follows: The radial pulsation apparently presents two stages, as if the ventricular system were duplicated. This shows on the tracing in the line of ascension, which, instead of being oblique and continuous, curves before it reaches the highest point into a hook, similar to that corresponding to the dicrotism in the descending line. In patients in whom he has met this pulse, the arterial system was almost normal, a condition which is apparently indispensable, in addition to very marked stenosis, for the production of this phenomenon. Potain remarked that the anacrotic pulse may exist in the absence of any lesion of the orifices of the heart, and he is therefore inclined to think that the heart valves have nothing to do with it. In other words, the wall of the heart being excessively distended, it undergoes two processes of emptying, resulting in the double rise which characterises the anacrotic pulse.

[5] N. Y. Med. Rec., Apr. 11, 1896. [6] Int. Med. Mag.; N. Y. Med. Rec., Mar. 28, 1896. [7] Deut. Arch. f. Kl. Med.; Quart. Journ., Apr., 1896. [8] Med. Chron., Apr., 1896. [9] Berl. Kl. Woch., 1896, No. 49; Am. Journ. of Med. Sc., Mar., 1896. [10] Sém. Méd., Mar. 14, 1896; Ep. Br. Med. Journ., Apr. 18, 1896. [11] Vratch, 1896, No. 4, p. 106; Les Nouv. Rém., Apr. 8, 1896. [12] Journ. des Pract., Mar. 28, 1896; N. Y. M. Journ., Apr. 11, 1896. [13] Les Nouv. Rémed, Jan. 24, 1896. [14] Edin. Med. Journ., June, 1896. [15] Med. Week., Apr. 24, 1896.

